# DNMT3a Deficiency Contributes to Anesthesia/Surgery‐Induced Synaptic Dysfunction and Cognitive Impairment in Aged Mice

**DOI:** 10.1111/acel.14458

**Published:** 2024-12-25

**Authors:** Peilin Cong, Xinwei Huang, Qian Zhang, Mengfan He, Hanxi Wan, Qianqian Wu, Huanghui Wu, Yuxin Zhang, Chun Cheng, Li Tian, Lize Xiong

**Affiliations:** ^1^ Shanghai Key Laboratory of Anesthesiology and Brain Functional Modulation, Clinical Research Center for Anesthesiology and Perioperative Medicine, Translational Research Institute of Brain and Brain‐Like Intelligence, Shanghai Fourth People's Hospital, School of Medicine Tongji University Shanghai China

**Keywords:** DNA methylation, DNMT3a, hippocampal neurons, perioperative neurocognitive disorder, synaptic

## Abstract

Perioperative neurocognitive disorder (PND) is a severe postoperative complication in older patients. Epigenetic changes are hallmarks of senescence and are closely associated with cognitive impairment. However, the effects of anesthesia and surgery on the aging brain's epigenetic regulatory mechanisms and its impact on cognitive impairment remain unclear. Using a laparotomy PND model, we report significant reduction in DNA methyltransferase 3a (DNMT3a) in hippocampal neurons of aged mice, which causes global DNA methylation decrease. Knockdown of DNMT3a leads to synaptic disorder and memory impairment in aged mice. Mechanistically, bisulfite sequencing revealed that DNMT3a deficiency reduces methylation in the LRG1 promoter region and promotes its transcription. We also show that activation of TGF‐β signaling by the increase in LRG1 level, ultimately impacts the synaptic function. In contrast, both overexpressing DNMT3a or knockdown LRG1 in hippocampus can attenuate the synaptic disorders and rescue postoperative cognitive deficits in aged mice. Our results reveal that DNMT3a is a previously undefined mediator in the pathogenesis of PND, which couples epigenetic regulations with anesthesia/surgery‐induced synaptic dysfunction and represents a therapeutic target to tackle PND.

## Graphical abstract

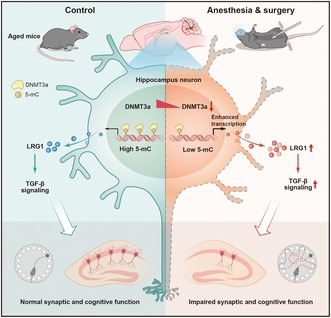

## 1 Introduction

Perioperative neurocognitive disorder (PND) is one of the most common perioperative complications (Mahanna‐Gabrielli et al. 2019), affecting up to 10.1%–59% of older patients undergoing surgery (Moller et al. 1998; Rappold et al. 2016). PND is associated with poor outcomes, including prolonged hospital stays, increased morbidity, and mortality (Witlox et al. 2010). With the unprecedented aging of the population, the burden of PND has increased substantially (Gou et al. 2021). However, the underlying mechanisms behind PND are still largely unknown.

Several studies have identified anesthetics, surgical stimuli, and aging as major risk factors for PND (Vutskits and Xie 2016). Mechanistically, epigenetic changes in brain cells occur during the senescent process, resulting in decreased resiliency and increased vulnerability (Crofts, Latorre‐Crespo, and Chandra 2024). When subjected to anesthetics and surgical stimuli, aging brain may experience exacerbated synaptic plasticity and cognitive function impairment. In this context, some epigenetic mechanisms (e.g., histone modifications and noncoding RNAs) have recently been implicated in rodent models of PND (Min et al. 2022; Wang, Shen, et al. 2024, Wang, Zhang, et al. 2024). Thus, understanding the underlying mechanisms of anesthesia/surgery on the epigenetic modifications in the aging brain will be beneficial to the development of early therapeutic strategies for PND.

DNA methylation is an epigenetic modification crucial for regulating gene expression. It can influence gene transcription through several mechanisms, such as modifying the binding of transcription factors and acting as docking sites for transcriptional repressors and corepressors (Parry, Rulands, and Reik 2021). Aberrant changes in neuronal DNA methylation patterns have been associated with postnatal growth and memory processes (Migliore and Coppedè 2022). Recent studies have found a decrease in global 5mC (5‐methylcytosine) levels in the plasma of patients undergoing breast surgery (Caputi et al. 2021). Some anesthetics (such as fentanyl and sevoflurane) and surgical stimuli can affect epigenetic function in mice brain (Lai et al. 2021; Sheng et al. 2022). These data suggest that DNA methylation may involve in the pathogenesis of anesthesia/surgery‐induced cognitive impairment.

DNA methyltransferases (DNMTs) are key enzymes that maintain DNA methylation, including DNMT1, DNMT3a, and DNMT3b. DNMT1 serves as the primary maintenance methyltransferase, preserving existing DNA methylation patterns. DNMT3a and DNMT3b are predominately responsible for the *de novo* methylation (Elliott et al. 2016). Knockout of DNMT3a or DNMT1 in mice causes neurological developmental disorders and typically die within 4 weeks of birth (Feng et al. 2010). Overexpression of DNMT3a enhances memory encoding by increasing the global DNA methylation and upregulating neuronal genes (*Arc* and *Bdnf*) (Su and Tsai 2012). However, whether anesthesia/surgery would induce DNMTs changes and participate in anesthesia/surgery‐induced cognitive impairment remains unclear.

In this study, we reported anesthesia/surgery caused a decrease in the levels of DNMT3a coincident with a reduction in global DNA methylation in hippocampus neurons. Combining DNA methylation sequencing and synaptic function analysis, we investigated the state of DNA methylation of functional regions regulating transcription. DNMT3a deficiency causes synaptic dysfunction and cognitive impairment by modulating 5mC at the promoter of leucine‐rich α2‐glycoprotein 1 (LRG1), upregulating LRG1 expression, and activating TGF‐β signaling. Furthermore, DNMT3a overexpression or LRG1 silencing alleviated anesthesia/surgery‐induced cognitive impairment. Our findings suggest that DNMT3a and DNA methylation alterations in the hippocampus play a causative role in PND and highlight potential regulatory targets for treatment.

## 2 Material and Methods

### 2.1 Animals

Male C57BL/6J mice (18–20 months) were purchased from Shanghai SIPPR‐BK Animal Co. Mice were housed in standard cages under controlled laboratory conditions (23°C ± 1°C, 50% humidity, 12‐h light–dark cycle with ad libitum access to food and water). All behavioral tests were performed during light phase. To avoid variability from the estrous cycle, only male mice were used. Each cage housed a maximum of four mice. Animal experiments were approved by the Ethics Committee on Experimental Animals of Tongji University (TJBH12423101).

### 2.2 Anesthesia/Surgery

Mice received laparotomy under isoflurane anesthesia using the methods described in our previous studies (Xu, Cong, Lu, et al. 2023). Mice were anesthetized with 1.4% isoflurane in 40% oxygen and placed on a heated table to maintain a temperature of 37.5°C. An abdominal incision was made, and the small intestine was kneaded for 10 min to mimic clinical laparotomy. The incision was closed in layers, and EMLA cream was used for analgesia. Each procedure lasted about 20 min. Mice then received 1.4% isoflurane in 40% oxygen for up to 2 h. Control group mice were placed in home cages with room air for 2 h, which is more clinically relevant.

### 2.3 Collection of Tissues

Mice were deeply anesthetized with isoflurane. We then harvested mouse hippocampus at 1, 3, or 7 days after anesthesia/surgery for the qPCR, western blot, and immunohistochemistry analysis.

### 2.4 Behavior Tests

#### 2.4.1 Open‐Field Test (OFT)

Each mouse was gently placed in an open‐field arena and allowed to explore freely for 5 min. All traces were recorded with a camera. The average speed and the time spent in the central area were quantified blindly using Smart Video Tracking Software (Panlab).

#### 2.4.2 Barnes Maze Test (BM)

Hippocampus‐dependent learning and memory were assessed through the BM as described previously (Xu, Cong, Zhang, et al. 2023). Mice explored an elevated circular platform with one escape holes. Training trials were performed repeatedly for 4 consecutive days. Latency to the target was measured. Probe test session was administered in which escape box was removed on fifth day. Time spent in target quadrant was calculated. Mice movement were recorded and analyzed with Smart Video Tracking Software (Panlab).

#### 2.4.3 Fear Conditioning (FC)

Mice were subjected to the contextual fear‐conditioning test described previously (Xu, Cong, Zhang, et al. 2023). Before training, mice freely explored the conditioning chamber for 10 min. During training, mice received five foot shocks (0.7 mA, 2 s, 35–60 s intervals). Contextual memory retrieval was assessed in the same chamber for 5 min. Freezing was defined as no movement for 2 s. Data were analyzed blindly using ANY‐maze Software. The apparatus was cleaned with 75% ethanol between each behavior test to eliminate odor cues.

### 2.5 S‐Adenosylmethionine (SAM) Treatment

SAM (Macklin Biochemical) was dissolved in saline. Each of the mice was injected with SAM solution in a dose of 100 mg/kg in 0.2 mL through intraperitoneal route 20 min before the anesthesia/surgery. An equal volume of saline was used as a control.

### 2.6 Stereotactic Injection

AAV2/9‐hsyn‐Dnmt3a‐3FLAG, AAV2/9‐hsyn‐EGFP‐U6‐shRNA‐Dnmt3a, AAV2/9‐hsyn‐EGFP‐U6‐shRNA‐Lrg1, and scramble virus were purchased from Biotech Shanghai. Virus were bilaterally injected into dorsal hippocampus (AP: −1.50 mm; ML: ±1.70 mm; DV: −1.75 mm). Three weeks later, mice were subjected to anesthesia/surgery or control conditions. After behavior tests, hippocampal tissue was harvested for immunofluorescence staining, qPCR, and western blotting.

### 2.7 RNA Sequencing

RNA sequencing was performed on hippocampal from control and surgery groups 1 day after surgery. cDNA libraries were constructed from 1 μg of total RNA using a cDNA‐PCR Sequencing Kit (SQK‐PCS109). The final cDNA libraries were subjected to FLO‐MIN109 flow cells and analyzed on the Oxford Nanopore PromethION platform. DEGs defined from the pairwise comparisons had to satisfy two selection criteria, including (i) FC ≥ 1.5 and (ii) adjusted *p* < 0.05.

### 2.8 Reduced Representation Bisulfite Sequencing (RRBS)

DNA was extracted using Qiagen extraction kit. RRBS libraries were prepared from 100 ng genomic DNA using the Acegen Rapid RRBS Library Prep Kit (AG0422, Acegen). Quality and concentration of RRBS libraries were determined using the Agilent 2100 Bioanalyzer and finally sequenced on Illumina novaseq 6000 platforms using a 150× 2 paired end‐sequencing protocol.

### 2.9 5‐mC and 5‐hmC Assay Assessment

Global DNA methylation and hydroxymethylation was quantified using a colorimetric ELISA kit (P1032 and P1030, Epigentek). DNA (100 ng) or 1 μL each standard was added to individual wells of a precoated 96‐well plate with 80 μL of binding buffer and incubated to allow DNA binding. Wells were washed and the primary anti‐5‐mC or anti‐5‐hmC added. Following incubation and washing, a detection antibody and enhancer solution were added and optical density was measured at 450 nm.

### 2.10 qPCR

Total RNA was extracted using the TRIzol and transcribed into cDNA using cDNA Synthesis Kit (Takara). qPCR analysis was conducted using TB Green Premix (Takara) on a Roche LightCycler 480 System (Roche). Primers were purchased from GenScript and sequences are shown in Table S1. The threshold cycle (Ct) values of the target genes were normalized to that of the housekeeping gene (endogenous control) encoding Gapdh. All data were analyzed by adopting Ct delta method (2^−ΔΔCt^).

### 2.11 Western Blot

The hippocampal tissues or cells were lysed in RIPA buffer (Thermo Fisher Scientific) containing 1 mM phosphatase inhibitor, 1 mM protease inhibitor, and 1 mM PMSF with an ultrasonicator. Total protein amounts were quantified using the Pierce BCA protein assay kit (Thermo Fisher Scientific). The proteins were separated on SDS–PAGE gels and then transferred onto a polyvinyl difluoride (PVDF) membrane (Millipore). The membrane was blocked with 3% BSA for 2 h at room temperature and then incubated with primary antibodies DNMT3a (ab188470, Abcam), LRG1 (13224, Proteintech), TGF‐β1 (AF1027, Affinity), SMAD2 (5339, CST), Phospho‐SMAD2 (18338, CST), SMAD3 (9523, CST), Phospho‐SMAD3 (9520, CST) overnight at 4°C followed by the HRP‐linked anti‐rabbit IgG antibody (31466, Thermo Fisher Scientific) for 1 h at room temperature. The target protein was detected by a ChemiDoc system (Bio‐Rad) and then analyzed with FIJI (ImageJ).

### 2.12 Immunohistochemistry

Mice were deeply anesthetized with isoflurane and transcardially perfused with 0.9% sodium chloride followed by 4% paraformaldehyde. Brains were removed, postfixed in 4% paraformaldehyde overnight, and then transferred to 30% sucrose until they sank. Frozen sections were prepared using a cryostat (Leica). For immunofluorescent staining, sections were washed in PBS and were blocked with 5% donkey serum and 0.5% Triton X‐100 for 120 min at room temperature. Primary antibodies 5‐mC (28692, CST), 5‐hmC (ab214728, Abcam), DNMT3a (20954, Proteintech), NeuN (MAB377, Milipore), GFAP (ab4674, Abcam), Iba1 (ab5076, Abcam), Flag (8146, CST), LRG1 (SC‐390920, Santa Cruz) were applied overnight at 4°C and secondary antibodies Donkey Anti‐Rabbit 594 (ab150064, Abcam), Donkey Anti‐Mouse Cy5 (AB_2340819, Jackson ImmunoResearch), Donkey Anti‐Chicken 488 (ab63507, Abcam), Donkey Anti‐Goat 488 (ab150129, Abcam) for 2 h at room temperature. Nuclei were labeled with DAPI (D9542, Sigma). Sections were finally observed and analyzed with Olympus FV2000 confocal microscope (Olympus) and FIJI (ImageJ).

### 2.13 Electrophysiological Recording

Electrophysiological recording was performed after behavioral tests as previously described (Xu, Cong, Zhang, et al. 2023; Zuo et al. 2021). Briefly, coronal slices containing the hippocampus (400 μm) were cut with a vibratome slicer (VT1200S, Leica) and incubated in artificial cerebrospinal fluid (ACSF) for at least 1 h before use. A single slice was transferred to the perfusion‐type recording chamber and visualized using infrared–differential interference contrast microscopy. A concentric bipolar electrode (FHC, Lot#300125) was positioned in the stratum radiatum of CA1 to stimulate the afferent Schaffer collateral–commissural pathway from the CA3–CA1 region. Then, fEPSPs were recorded in the CA1 region using micropipettes and LTP was induced by delivery of HFS (100 Hz, 20‐s interval, four trains), the amplitude of the fEPSPs was usually set at 40%–50% of the maximal responses. Data were recorded with a MultiClamp 700B (Axon instruments) and acquired with Clampex 10.7.

### 2.14 Golgi Staining

Mice brain was removed after the behavioral tests and immediately fixed in 30% sucrose solution for 48 h. Then, the brain was treated with a Golgi staining kit (g1069, Servicebio) for 5 days. After staining, tissue blocks were cut into 50‐μm‐thick sections, and then sections were dehydrated in absolute ethanol twice for 20 min and cleared in xylene for 30 min. Images of dendritic spines were acquired using a 60× objective, and hippocampal CA1 pyramidal neurons were selected for analysis. Sholl analysis were performed in FIJI (ImageJ).

### 2.15 Chromatin Immunoprecipitation Assay

Chromatin immunoprecipitation (ChIP) assays were detected using a ChIP kit (P‐2002, Epigentek). Briefly, hippocampal tissues were cross linked with 1% formaldehyde, and then sonicated in ChIP lysis buffer to shear DNA. Chromatin–protein complexes were immunoprecipitated with anti‐DNMT3A (20954, Proteintech). An antibody against normal IgG was used as a negative control. Protein‐free DNA was extracted and the relative abundance of the target at the promoter was quantified by RT‐qPCR targeting the Lrg1 promoter locus. Primers are listed in Table S1. The target values were normalized to the input controls.

### 2.16 Cell Culture

Neuro‐2a (N2a) cells were obtained from the American‐Type Culture Collection (ATCC). The cells were cultured in Dulbecco's modified Eagle's medium (DMEM) supplemented with 10% (v/v) fetal bovine serum (Gibco), 100 μg/mL streptomycin, and 100 units/mL penicillin. Cells were cultured at 37°C with 5% (v/v) CO_2_.

### 2.17 Gene Silencing of DNMT3a in Cells

Lentivirus for silencing Dnmt3a expression in N2a cell was purchased from Biotech Shanghai. N2a cells were seeded into a six‐well plate 24 h prior to lentiviral infection. On the day of infection, the culture media was removed from the wells and replaced with 1 mL of poly/media mixture. Lentiviral particles of DNMT3a shRNA or control shRNA were thawed at room temperature and incubated with cells overnight. Cells were expanded and assayed for stable shRNA expression 48 h after transfection.

### 2.18 Determination of LRG1

Mice were anesthetized and perfused with 0.9% saline, followed by dissection of the hippocampal tissues. Cell medium was collected following DNMT3a knockdown in N2a cells. The concentrations of LRG1 were measured using ELISA kits (OKCD01614, Aviva Systems Biology), following the manufacturer's instructions. Each sample was analyzed in triplicate to ensure accuracy and reproducibility.

### 2.19 Targeted Bisulfite Sequencing

Gene‐specific DNA methylation was assessed by a next‐generation sequencing based BSP, according to our previously published method. BSP primers were designed using the online MethPrimer software and listed in Table S1. For each sample, BSP products of multiple genes were pooled equally, 5′ phosphorylated, 3′ dA tailed and ligated to barcoded adapter using T4 DNA ligase (NEB). Barcoded libraries from all samples were sequenced on Illumina platform.

### 2.20 Statistical Analysis

The data are shown as the mean ± SEM, with the presentation of data of each individual animal. We did not exclude any data. All the assays were repeated at least three times. Shapiro–Wilk test was used to check whether the data were normally distributed. Student's *t* test (two groups), one‐way ANOVA followed by Dunnett's post hoc test (multiple groups), and repeated measures two‐way ANOVA followed by Sidak's post hoc test (multiple groups at different time point) were used to analyze the data using GraphPad Prism 8.0 software (GraphPad Prism Co., USA). Data that did not exhibit a normal distribution were analyzed via Kruskal–Wallis test (multiple groups); *p* < 0.05 was considered significant.

## 3 Results

### 3.1 Anesthesia/Surgery Decreased DNA Methylation in the Hippocampus of Aged Mice

We used the laparotomy model under isoflurane in aged mice. Cognitive function was observed as outlined in Figure S1A. There were no significant differences in the average speed and time spent in the central zone between the groups. We assessed learning and memory behavior using the Barnes maze and fear conditioning on days 3–10 after surgery. Mice showed hippocampus‐dependent cognitive impairment (Figure S1B–H).

**FIGURE 1:**
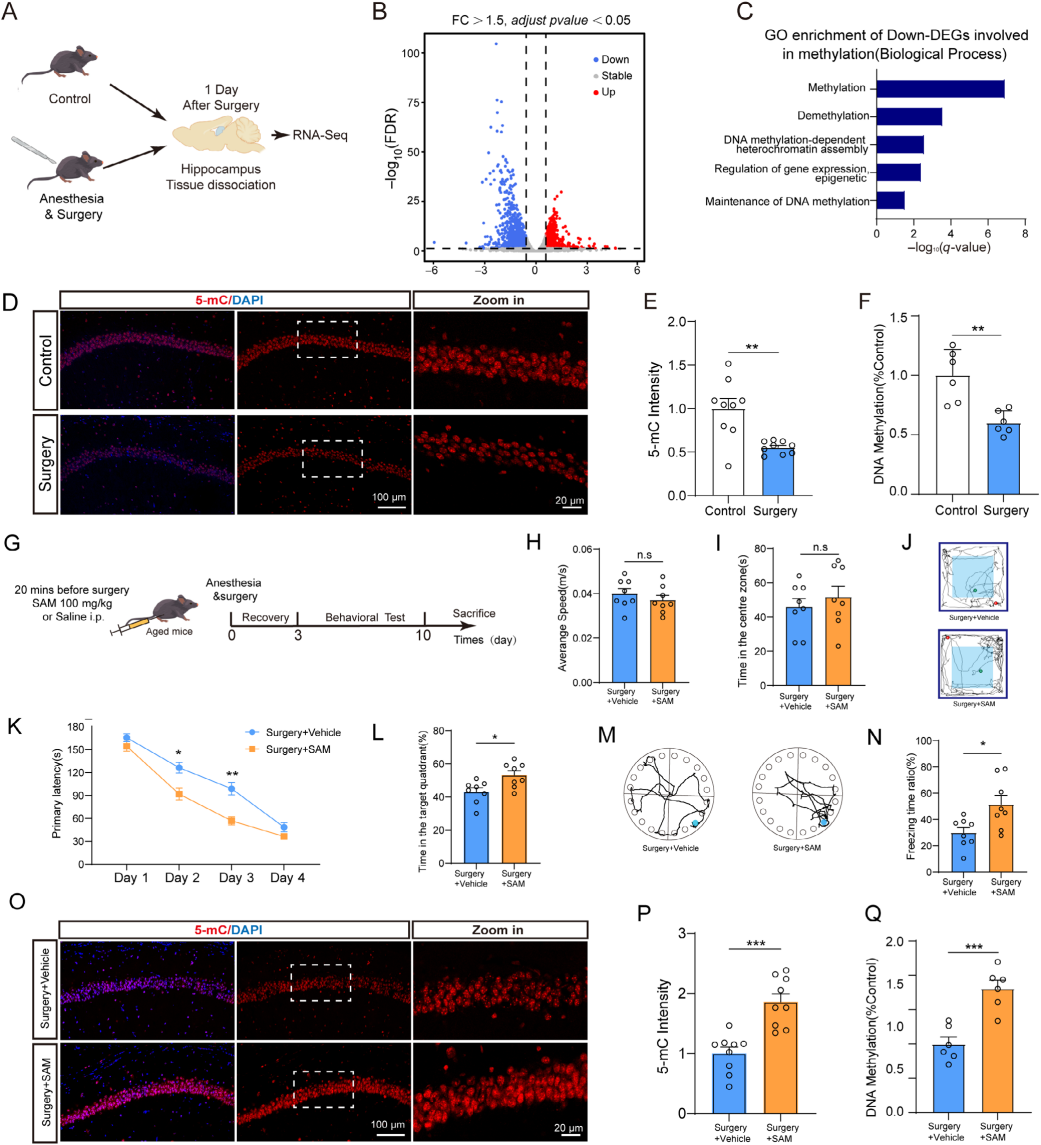
Anesthesia/surgery reduced 5‐mC levels in the hippocampus of aged mice. (A) Experimental design for RNA‐Seq. (B) Volcano plot showing differentially regulated genes. (C) Gene ontology analysis of downregulated genes are involved in methylation modification. (D, E) Representative field and quantification of 5‐mC expression in the hippocampus (*n* = 9). (F) Global DNA methylation level in hippocampus (*n* = 6). (G) Experimental design for SAM treatment. Mice received SAM or vehicle intraperitoneal injection. (H) Average speed, (I) time in the central area, and (J) representative movement tracks in the OFT (*n* = 8). (K) The escape latency. (L) The percentage of time spent in the target quadrant and (M) representative mouse movement tracks in BM (*n* = 8). (N) Percentage of freezing times (*n* = 8). (O, P) Representative field and quantification of 5‐mC expression in the hippocampus (*n* = 9). (Q) Global DNA methylation level in the hippocampus (*n* = 6). All values are presented as mean ± SEM (*p < 0.05, **p < 0.01, and ***p < 0.001, unpaired *t* test for E, F, H, I, L, N, P, Q and two‐way ANOVA with Bonferroni post hoc test for K).

To explore the underlying molecular and the related changes in cellular function after anesthesia and surgery, we first performed RNA‐seq 1 day after surgery (Figure 1A). This time point was selected as it represents a pivotal window for identifying molecular alterations underlying the development of PND and is widely recognized as an optimal period for diagnostic and prognostic biomarker discovery (Chen et al. 2022; McKay et al. 2022). Our preliminary experiments also demonstrated that mice exhibited significant learning and memory deficits as early as postoperative day 1 (Figure S2A–D). The PCA and heatmap revealed the separation of samples between control and surgery mice (Figure S2E,F). We identified 2596 differentially expressed genes (FDR < 0.05 and fold change > 1.5), with 953 genes upregulated and 1643 genes downregulated (Figure 1B; Table S2). Gene ontology (GO) revealed a highly significant enrichment in DNA methylation, including methylation, demethylation, and DNA methylation maintenance (Figure 1C). As DNA methylation and demethylation are the two main types of methylation functions that preserve DNA methylation homeostasis, we examined changes in 5‐mC (marker for DNA methylation) and 5‐hmC (marker for DNA demethylation) levels after surgery and observed a significant decrease in 5‐mC levels (Figure 1D–F). In contrast, DNA demethylation was unaffected in the hippocampus, as illustrated by no significant changes in 5‐hmC (Figure S3A–C). Additionally, by treating mice with SAM (a methyl donor) (Gregoire et al. 2017), we found that SAM alleviated anesthesia/surgery‐induced cognitive impairment and improved DNA methylation levels (Figure 1J–P). These data suggested that decreased DNA methylation after surgery plays an important role in PND pathogenesis.

### 3.2 Decreased 5‐mC Is Correlated With Reduced Expression of DNMT3a in Hippocampal Neurons After Anesthesia/Surgery

**FIGURE 2:**
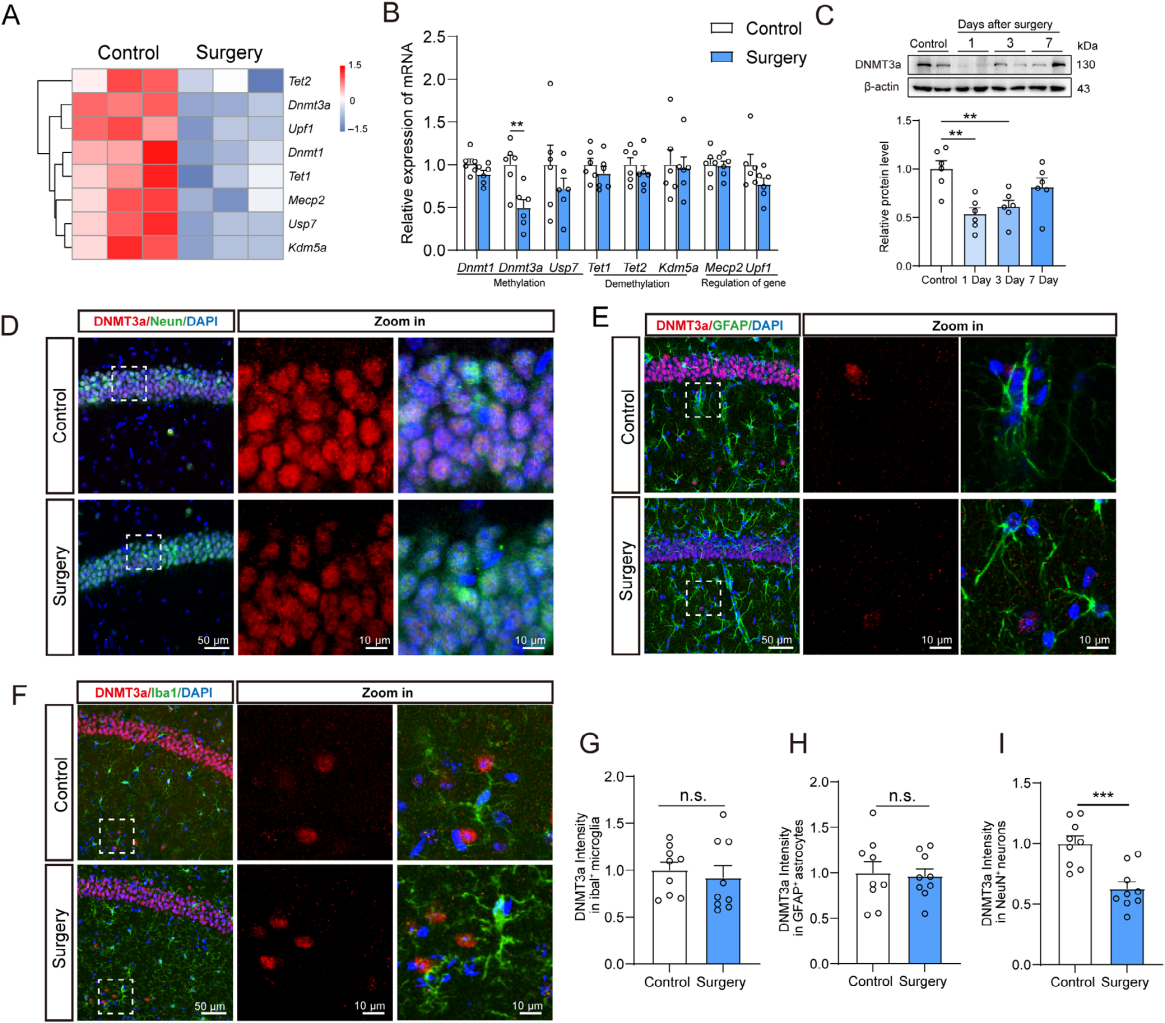
Anesthesia/surgery decreased the expression of DNMT3a mRNA and protein in hippocampal neurons. (A) Heatmap of genes associated with DNA methylation. (B) Relative expression of genes associated with DNA methylation from hippocampal (*n* = 5). (C) Protein blotting bands for DNMT3a in hippocampus tissues and quantification of relative protein expression (*n* = 6). Representative images of DNMT3a co‐stained with different cell types. (D) Neurons (NeuN) or (E) astrocytes (GFAP) or (F) microglia (Iba1) in the hippocampal CA1 region. Quantification of DNMT3a intensity in different types of cells: (G) microglia or (H) astrocytes or (I) neurons (*n* = 9). All values are presented as mean ± SEM ( **p < 0.01, and ***p < 0.001, unpaired *t* test for B, G, H, I and one‐way ANOVA with Dunnett's test for C).

Next, we examined how the 5‐mC level is downregulated after surgery. To this end, we first identified genes associated with DNA methylation (Figure 2A). We confirmed this by qPCR and found anesthesia/surgery mainly led to a decrease in the mRNA level of DNMT3a (Figure 2B). Western blot revealed a significant decline in DNMT3a expression following surgery, which subsequently showed a gradual recovery until seventh day after surgery (Figure 2C). DNMT3a is mainly expressed in the CNS and is crucial for embryonic and postnatal brain development and memory formation in adulthood (Su and Tsai 2012). To examine changes in DNMT3a expression in different brain cell types, we performed immunofluorescence costaining of DNMT3a along with antibodies against markers for neurons (NeuN), astrocytes (GFAP), and microglia (Iba1). DNMT3a was coexpressed with NeuN (Figure 2D). We did not observe obvious differences in DNMT3a signal intensity in microglia or astrocytes between the control and surgery groups (Figure 2E,F). However, DNMT3a intensity significantly declined in hippocampal neurons (Figure 2G–I). Altogether, these data raise the possibility that decreased DNMT3a in neurons is correlated with reduction in the 5‐mC level in the hippocampus and may be involved in anesthesia/surgery‐induced cognitive impairment in aged mice.

### 3.3 Reduction of DNMT3a Induces Synaptic Disorders and Cognitive Impairment in Aged Mice

**FIGURE 3:**
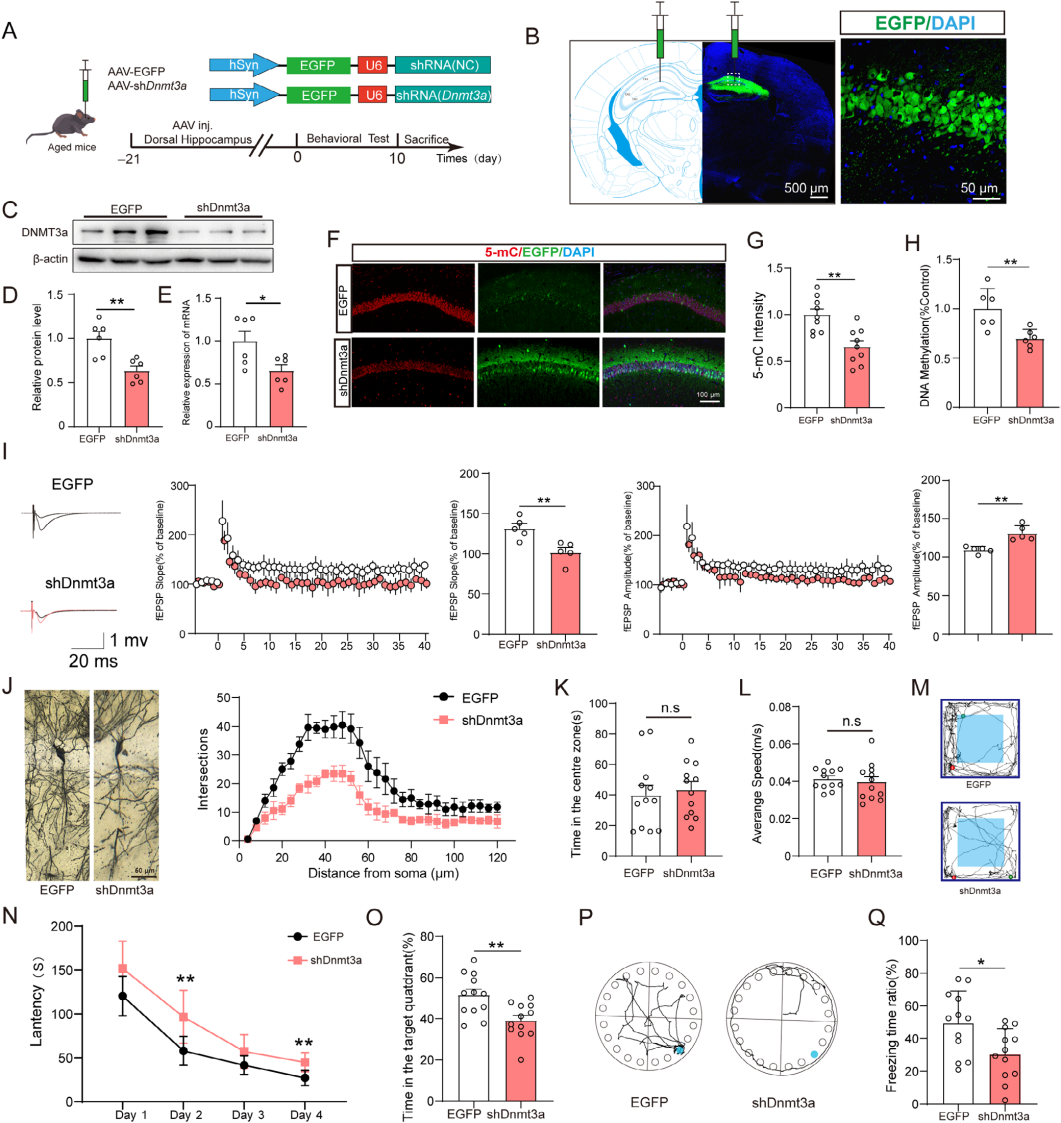
DNMT3a knockdown in the hippocampus leads to decreased DNA methylation levels, synaptic disorder, and memory deficiency. (A) Schematic of the experimental paradigm. (B) Representative fluorescence image of the AAV‐infected slice. (C) Protein blotting bands for DNMT3a. (D) Quantification of relative protein expression. (E) Relative gene expression in hippocampus (*n* = 6). (F, G) Representative field and quantification of 5‐mC expression in the CA1 (*n* = 9). (H) Global DNA methylation level in the hippocampus (*n* = 6). (I) Normalized the fEPSP slope and amplitude at hippocampal. Quantitative analysis of fEPSP slope and amplitude at last 20 min (*n* = 5). (J) Golgi staining images showing the dendritic trees in hippocampus. The Sholl analysis was performed to evaluate the dendritic complexity (*n* = 5). The average speed (K), time spent in the central area (L), and representative movement track (M) in OFT (*n* = 12). The escape latency over the training session (N). The percentage of times spent in the target quadrant (O) and representative movement tracks (P) (*n* = 12). (Q) The percentage of freezing times (*n* = 12). All values are presented as mean ± SEM (*p < 0.05, **p < 0.01, unpaired *t*‐test for D, E, G, H, I, K, L, O, Q and two‐way ANOVA with Bonferroni post hoc test for J).

We aimed to assess the roles of DNMT3a in the pathogenesis of surgery‐induced cognitive impairment. We constructed a DNMT3a‐knockdown vector with a hSyn promoter to specifically knockdown DNMT3a expression (Figure 3A,B). Western blotting and qPCR suggested that this knockdown strategy was successful (Figure 3C–E). In parallel, we found that the 5‐mC level significantly decreased during DNMT3a knockdown (Figure 3F–H). Since synaptic plasticity in the hippocampus is widely considered the basis of learning and memory, we examined changes in synaptic plasticity in shDNMT3a‐treated mice. Our assessment of the long‐term potentiation (LTP) at hippocampal revealed that shDNMT3a‐infected mice exhibited impaired normalized field excitatory postsynaptic potentials (fEPSP) slope and amplitude (Figure 3I). In addition, Golgi staining revealed that shDNMT3a reduced the density of dendritic spines (Figure 3J). These findings suggest that loss of DNMT3a expression could lead to synaptic dysfunction in aged mice, which was also observed in the PND mouse model (Xu, Cong, Zhang, et al. 2023). Next, we examined whether the loss of DNMT3a affects cognitive function in aged mice. DNMT3a knockdown did not affect motion capabilities (Figure 3K–M). However, shDNMT3a‐injected mice displayed longer target‐hole latency and reduced target‐search time in the BM test (Figure 3N–P). In the FC test, shDNMT3a‐infected mice showed a significant decrease in freezing time in the contextual conditioning paradigms (Figure 3Q). Our results suggest that loss of DNMT3a expression could induce cognitive impairment in aged mice and plays an important role in PND‐associated pathologies.

### 3.4 Anesthesia/Surgery‐Induced Changes in Hippocampal DNA Methylation Profiles in Aged Mice

**FIGURE 4:**
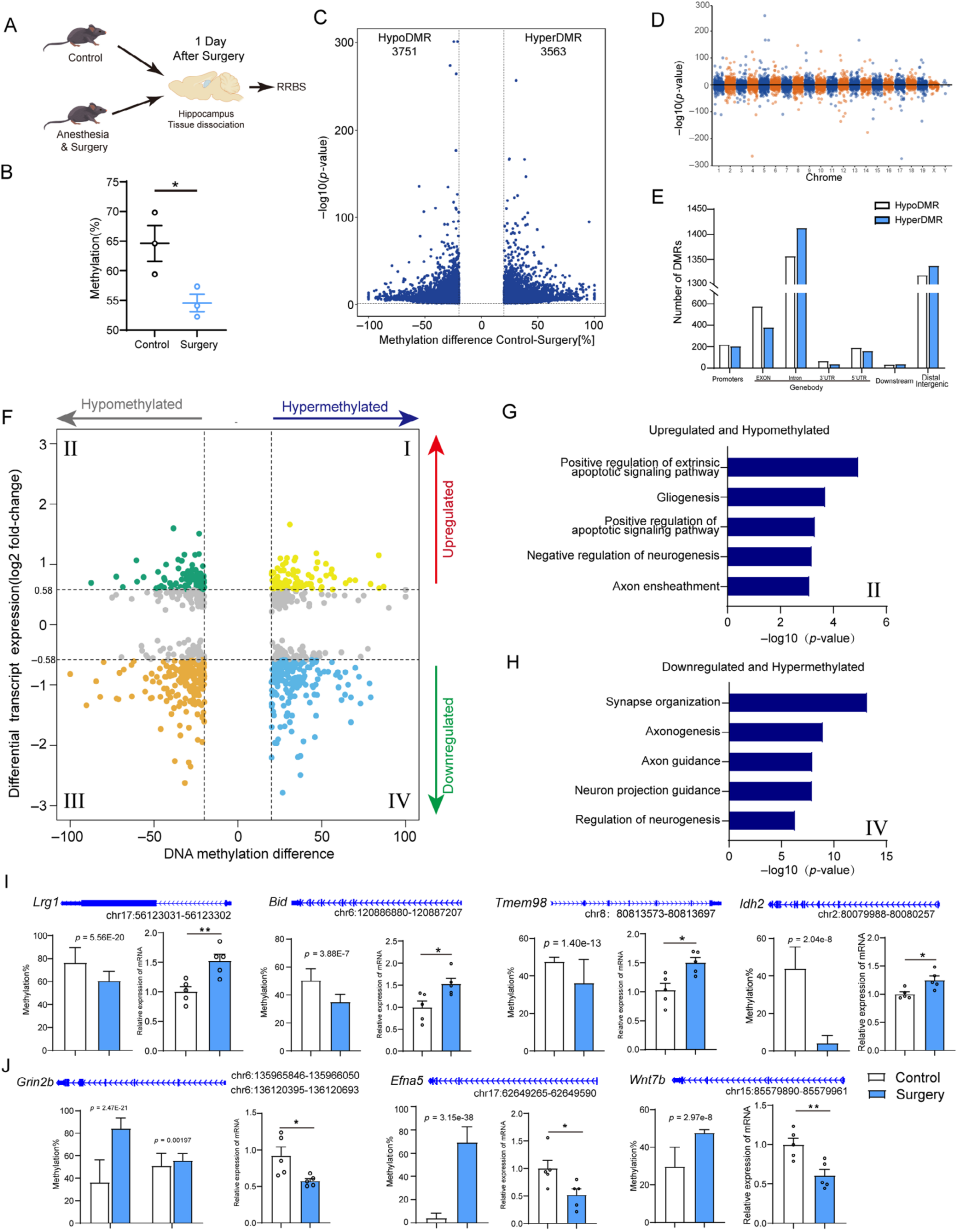
DNA methylation and transcript change after anesthesia/surgery in the hippocampus of aged mice. (A) Scheme of the experimental approach used for RRBS. (B) Total methylation levels of CG sites in RRBS from the hippocampus in the control and surgery groups (*n* = 3). (C) Volcano plot of surgery‐induced differentially methylated regions. (D) Manhattan plot depicting the distribution of DMRs over chromosomes. (E) Genomic feature distributions of differential DNA methylation regions. (F) Quadrant plot of DMRs and differentially expressed genes. The *x*‐axis indicates differences in DNA methylation. The *y*‐axis indicates a log2‐fold change in transcript levels. Horizontal (20% difference) and vertical (1.5‐fold change) dashed lines show four quadrants: (I) hypermethylated and upregulated genes, (II) hypomethylated and upregulated genes, (III) hypomethylated and downregulated genes, and (IV) hypermethylated and downregulated genes. Top GO categories for upregulated and hypomethylated genes (G) and downregulated and hypermethylated genes (H). Average DNA methylation levels and mRNA levels of the candidate gene. Note the significantly hypomethylated and upregulated genes (I) and the hypermethylated and downregulated genes (J). Data indicate mean ± SEM. *p < 0.05; **p < 0.01 (unpaired *t* test for B, I, J).

DNA methylation can affect transcriptional activation. To generate a genome‐wide map of the DNA methylation profiles affected by anesthesia/surgery in aged mice, we performed RRBS between the control and surgery groups on the same hippocampal tissue in the RNA‐Seq experiment (Figure 4A). Among these, we identified 143,109 differentially methylated CpGs (*q* < 0.05), with 73,588 hypomethylated and 69,521 hypermethylated CpGs. The average CpG methylation levels revealed global DNA hypomethylation in the interrogated sequences in the surgery group compared with that in the control group (Figure 4B), which is consistent with our observations in Figure 1.

Regions containing at least three CpGs with a minimum difference of 20% were classified as differentially methylated regions (DMRs). This revealed the clustering of CpGs into 7314 DMRs, with 3563 hypermethylated regions and 3751 hypomethylated regions (Figure 4C and Table S3). DMRs were distributed across all chromosomes in the genome (Figure 4D) at different genomic locations. Overall, hypoDMGs were relatively more enriched in the promoter and exon regions than hyperDMGs, whereas hypermethylation occurred more in the intron and distal intergenic regions (Figure 4E). Given the difficulty in linking DNA methylation changes at intergenic regions to their target genes, we limited this analysis to the promoter region and gene body.

Furthermore, to link DNA methylation states to gene expression affected by anesthesia/surgery in aged mice (refer to Figure 1B), we visually presented this analysis through a quadrant plot (Figure 4F). Given that most genes exhibit an inverse correlation between DNA methylation and gene expression, we mainly focused on Quadrant II (Table S4) and Quadrant IV (Table S5). Quadrant II included hypomethylated genes with increased expression after surgery, which included genes related to cell apoptosis (e.g., *Lrg1*, *Bid*) (Hung et al. 2021; Ruan et al. 2023) and axon ensheathment (e.g., *Tmem98*, *Idh2*) (Huang et al. 2018) (Figure 4G,I). Quadrant IV included hypermethylated genes with decreased expression, which included genes related to synapse organization (e.g., *Grin2b*, *Wnt7b*) (Fazel Darbandi et al. 2020; Tullis et al. 2023) and axonogenesis (e.g., *Efna5*) (Chai et al. 2014) (Figure 4H,J). Supporting these findings, qPCR demonstrated changes in the hippocampus, suggesting a potential relationship between DNA methylation and gene expression. Altogether, these results demonstrated that there were DNA methylation alterations during surgery in the hippocampus of aged mice, which included genes involved in maintaining synaptic and neuronal functions.

### 3.5 DNMT3a Deficiency Represses LRG1 Gene Expression and Activates TGF‐β Signaling

**FIGURE 5:**
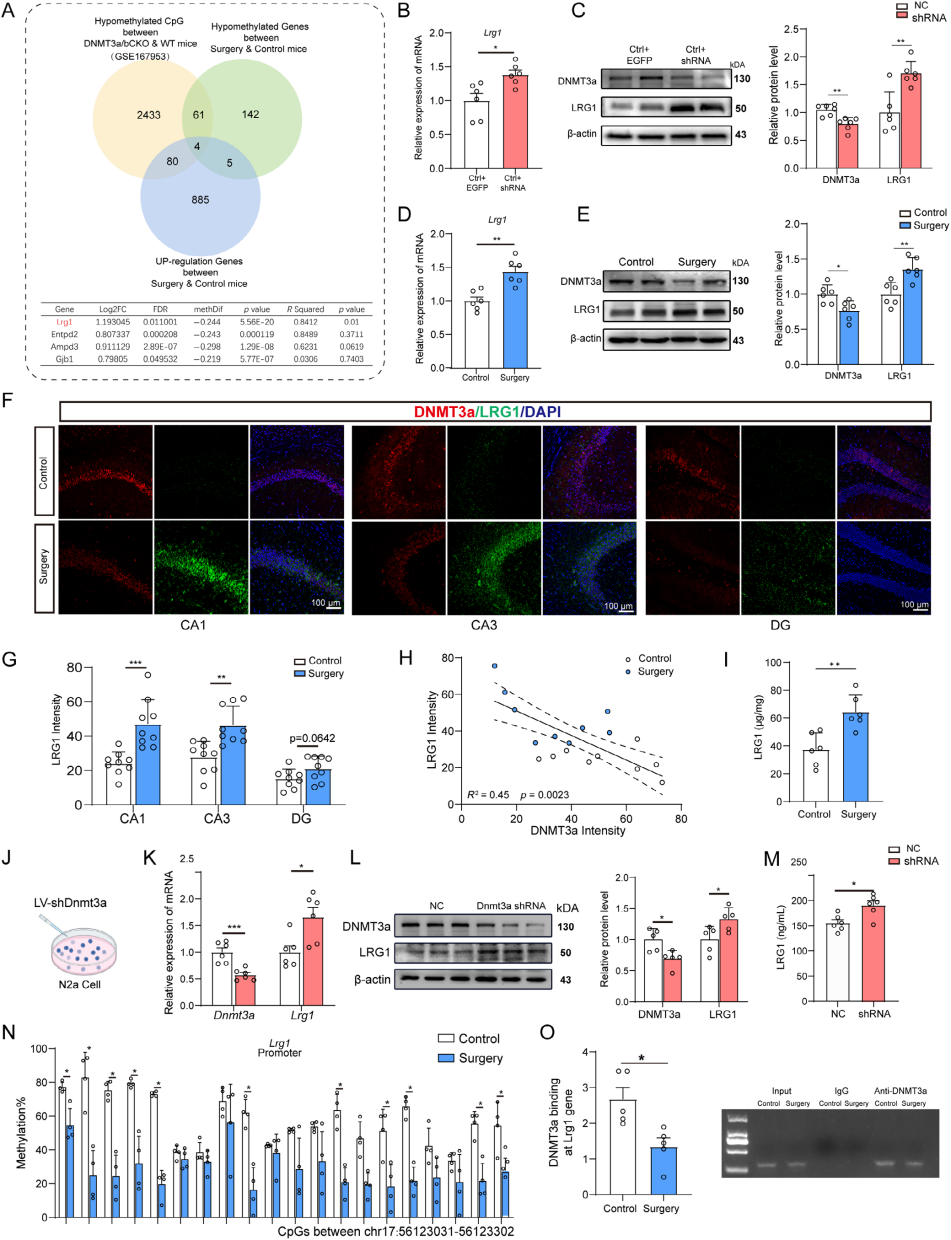
Loss of DNMT3a results in hypomethylation and upregulation of LRG1. (A) Venn diagram and correlation for the identified genes. Correlation between mRNA expression and DNA methylation level of four overlap genes (table). Relative gene expression of LRG1 in the hippocampus (*n* = 6) (B), protein blotting bands and quantification of the relative protein expression of LRG1 in the hippocampus of EGFP or shDNMT3a AAV‐injected mice (C) (*n* = 6). Relative gene expression of LRG1 in the hippocampus (D) (*n* = 6), protein blotting bands and quantification of relative protein expression for LRG1 between the control and surgery groups (E) (*n* = 6). (F) Representative field of DNMT3a (red) and LRG1 (green) in the hippocampal of mice in control and surgery groups. (G) Quantification of LRG1 expression in the hippocampus CA1, CA3, and DG (*n* = 9). (H) Correlation analysis of intensity between DNMT3a and LRG1 in CA1. (I) LRG1 concentration in the hippocampal tissue between control and surgery group (*n* = 6). (J) Schematic of the experimental paradigm. N2a received LV‐shRNA (Dnmt3a) or NC. (K) Relative gene expression of DNMT3a and LRG1 between the NC and shRNA groups (*n* = 6). (L) Protein blotting bands for DNMT3a and LRG1 between the NC and shRNA groups. Quantification of relative protein expression (*n* = 6). (M) LRG1 concentration in the N2a cell medium (*n* = 6). (N) Validation of surgery‐induced CpG methylation changes at the promoter region of LRG1 (chr17:56123031–56123302) using targeted bisulfite sequencing of the hippocampus tissue in the control and surgery groups (*n* = 3). (O) ChIP‐qPCR analysis demonstrating DNMT3a binding to the LRG1 promoter (*n* = 5). Data are presented as mean ± SEM; two‐tailed *t* tests were used unless otherwise specified. *p < 0.05, **p < 0.01, ***p < 0.001 versus EGFP group.

Next, we sought to dissect the mechanism by which DNMT3a in the hippocampus contributes to synaptic disorder and cognitive impairment after surgery. We reanalyzed the published sequencing data of DNMT3a/b‐CKO in hippocampal neuron (Zocher et al. 2021). We only considered DMRs that overlapped with the promoter region. Four genes coincided with surgery‐induced upregulation, with Lrg1 being of particular interest. LRG1 modulates TGF‐β signaling in a tissue‐specific manner (Wang et al. 2013) and has been implicated in the impairment of synaptic transmission (Liu et al. 2022). RRBS showed that surgery could cause a significant decrease in the DNA methylation level of the LRG1 promoter region (chr17:56123031–56123302) and significantly increase LRG1 gene expression. Pearson's correlation analysis showed that the expression and methylation levels of LRG1 were significantly associated (Figure 5A). We predicted that the increased expression of LRG1 was regulated by a decrease in DNMT3a. To test this hypothesis, we further examined the relationship between DNMT3a and LRG1. We found that the injection of shRNA‐Dnmt3a significantly decreased LRG1 mRNA and protein levels in the hippocampal tissue of aged mice (Figure 5B,C). Similarly, anesthesia/surgery upregulated expression of LRG1 mRNA and protein in the hippocampus of aged mice (Figure 5D,E). Furthermore, immunofluorescence analysis revealed a significant increase in LRG1 fluorescence intensity in the CA1 and CA3 regions after anesthesia and surgery (Figure 5F,G). An increase was also observed in the DG region, though this change was not statistically significant. Notably, DNMT3A and LRG1 showed a colocalization in hippocampus, suggesting a potential interaction between them. Correlation analysis further indicated that the decrease in DNMT3A expression was negatively correlated with LRG1 levels (Figure 5H). As LRG1 is a secreted molecule that can exert autocrine and paracrine effects, we utilized ELISA to measure the concentration of LRG1 in hippocampal tissues. The results showed that the LRG1 concentration increased by approximately 1.85‐fold following anesthesia and surgery (Figure 5I). To elucidate the regulatory relationship between DNMT3a and LRG1 in neuron, we further checked the LRG1 in N2a cells. DNMT3a ablation by LV‐shRNA increased LRG1 mRNA and protein expression in N2a cells (Figure 5J–L). Additionally, such upregulation was observed in N2a cell medium (Figure 5M), suggesting excessive neuronal release. The described evidence strongly suggests the participation of DNMT3a in surgery‐induced LRG1 upregulation in the hippocampus. To further define the mechanisms by which DNMT3a regulates the transcription of LRG1 gene, we performed TBS for the CpG sites located within approximately 271 bp in the promoter region of LRG1 and found a general decrease in the DNA methylation level (Figure 5N). Chromatin immunoprecipitation (ChIP) revealed that the binding of DNMT3a to LRG1 promoter region was reduced in the surgery group (Figure 5O). Because LRG1 modulates TGF‐β signaling in a tissue‐specific manner, it has been widely reported that TGF‐β is required for brain homeostasis and maintain cognitive function in mice (Bedolla et al. 2024; Wang et al. 2023). We further examined TGF‐β signaling molecules. Western blotting showed that DNMT3a knockdown enhanced the protein levels of TGF‐β1. Meanwhile, DNMT3a knockdown did not affect SMAD2 and SMAD3, but increased both phosphorylated SMAD2 (pSMAD2) and phosphorylated SMAD3 (pSMAD3) (Figure S4A). Similar results were also found between the control and surgery groups (Figure S4B). Overall, these findings suggested that loss of DNMT3a could decrease the DNA methylation of LRG1, upregulate the expression of LRG1, and activate TGF‐β signaling in aged mice.

### 3.6 Overexpression of DNMT3a Rescues Anesthesia/Surgery‐Induced Changes in Aged Mice

**FIGURE 6:**
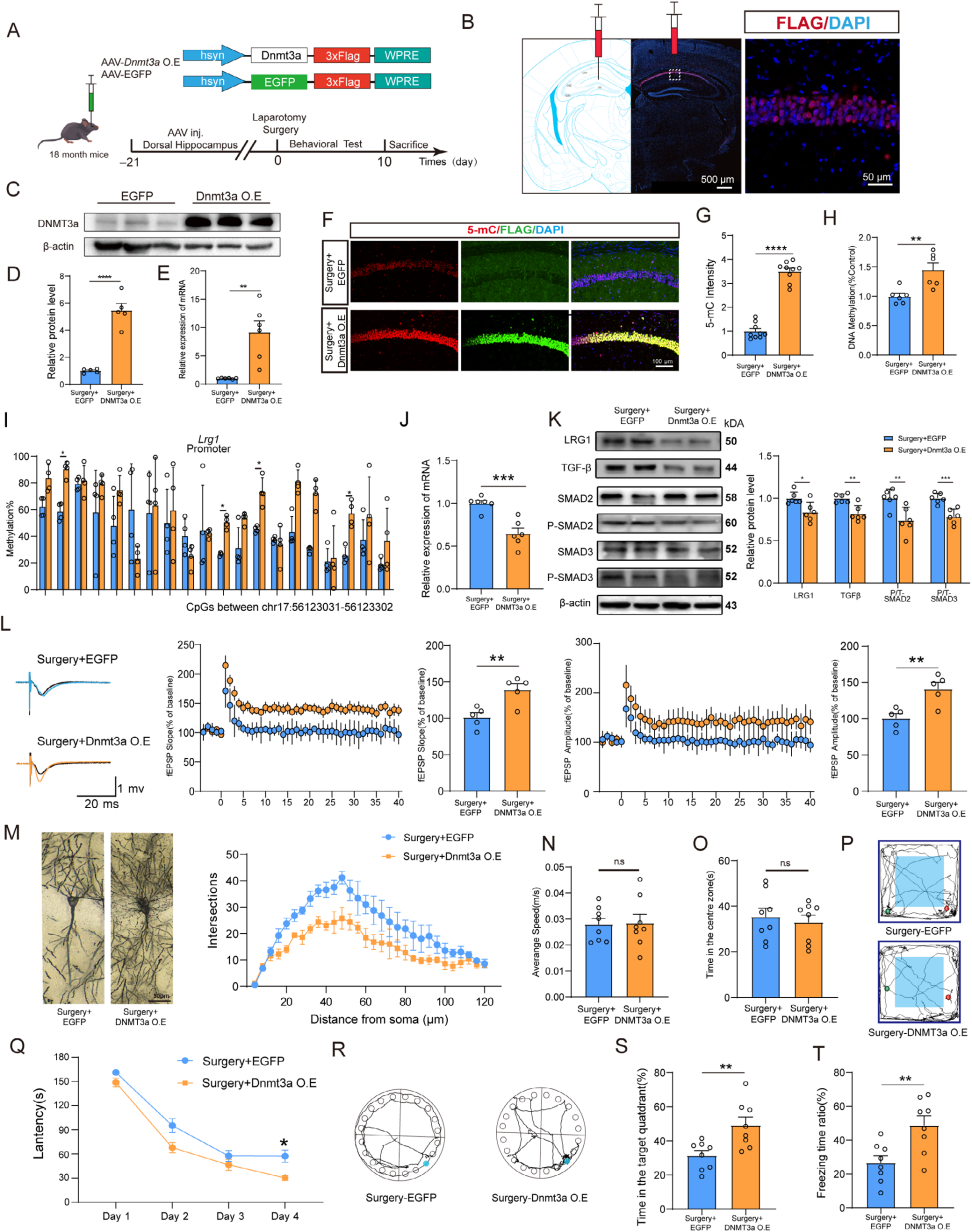
Overexpression of DNMT3a rescues cognitive impairment and synaptic deficits after anesthesia/surgery. (A) Schematic of the experimental paradigm and diagram of the AAV. (B) Representative fluorescence image of the virus‐infected slice. (C) Protein blotting bands for DNMT3a. (D) Quantification of relative protein expression. (E) Relative gene expression of DNMT3a in hippocampal. (F, G) Representative field and quantification of 5‐mC expression in CA1. (H) Global DNA methylation level in the hippocampus (*n* = 6). (I) Validation of surgery‐induced CpG methylation changes in the promoter region of LRG1 (chr17:56123031–56123302) (*n* = 4). (J) Relative gene expression of LRG1 in the hippocampus (*n* = 5). (K) Protein blotting bands quantification of relative protein expression for LRG1, TGF‐β1, SMAD2, p‐SMAD2, SMAD3, and p‐SMAD3 (*n* = 6). (L) The normalized fEPSP slope and amplitude at hippocampal. Quantitative analysis of fEPSP slope and amplitude in the last 20 min (*n* = 5). (M) Golgi staining images showing dendritic trees in the hippocampus. Sholl analysis was performed to evaluate the dendritic complexity (*n* = 5). The average speed (N), time spent in the central area (O), and representative movement tracks (P) in the open‐field test (*n* = 8). The escape latency over the training session (Q). The representative movement tracks (R) and percentage of times spent in the target quadrant (S) (*n* = 8). (T) Percentage of freezing times (*n* = 8). All values are presented as mean ± SEM (*p < 0.05, **p < 0.01, and ***p < 0.001, unpaired *t* test for D, E, G, H, I, J, K, N, O, S, T and two‐way ANOVA with Bonferroni post hoc test for M and Q).

Next, we explored whether DNMT3a overexpression alleviates synaptic damage and cognitive impairment induced by surgery in aged mice. We injected AAV‐DNMT3a with an hSyn promoter (Figure 6A,B). Overexpression of DNMT3a was confirmed by western blotting and immunostaining (Figure 6C–E). We found that DNMT3a overexpression could not only enhance the global 5‐mC level in the hippocampus (Figure 6F–H), but also partly rescue changes in DMRs in the promoter region of LRG1 induced by surgery (Figure 6I). Meanwhile, surgery‐induced upregulation of LRG1 and activation of TGF‐β signaling was reversed by DNMT3a‐overexpressing (Figure 6J–K). Moreover, the surgery‐induced LTP deficit and density of dendritic spines decreased in aged mice and could also be partially rescued (Figure 6L–M). The OPF, BM, and FC tests revealed significant improvements in learning and memory, notably reduced latency to mount a target hole, increased time spent in the target quadrant, and prolonged freezing time in DNMT3a‐overexpressing mice (Figure 6N–T). Taken together, these findings demonstrated that DNMT3a overexpression in the hippocampus could regulate LRG1/TGF‐β loop and alleviate synaptic loss and cognitive impairment in aged mice.

### 3.7 Knockdown of Lrg1 Rescues Surgery‐Induced Memory Impairment and Synaptic Disorders in Aged Mice

Although the induction of LRG1 was previously reported in the regulation of learning and memory in aged mice, the role of LRG1 in PND remains unknown. We then investigated whether LRG1‐mediated therapy can attenuate surgery‐induced cognitive decline in aged mice using a neuron, specifically AAV‐shRNALrg1 (Figure S5A,B). LRG1 knockdown efficiency was confirmed by western blotting and qPCR (Figure S5C–E). Consistent with our expectations, knockdown of LRG1 significantly inhibited the activation of the TGF‐β pathway after surgery (Figure S5F). In addition, we found that LRG1 knockdown could partially rescue the surgery‐induced LTP deficit in the hippocampus of aged mice (Figure S5G). Behavioral tests revealed that LRG1 knockdown rescued surgery‐induced cognitive deficits in aged mice (Figure S5K–N) without affecting locomotor activity (Figure S5H–J). These results demonstrate that interruption of the DNMT3a/LRG1/TGF‐β loop in the hippocampus can be considered a potential strategy for alleviating PND.

## 4 Discussion

In this study, we identified DNMT3a as a pivotal functional DNA methyltransferase involved in PND. DNMT3a downregulation appears to causally contribute to surgery‐induced cognitive impairment, whereas its overexpression effectively alleviated cognitive impairment behaviors. Mechanistically, DNMT3a downregulation induced by anesthesia and surgery could disrupt DNA methylation stability in the hippocampus of aged mice. This decrease could modulate 5mC at the promoter of LRG1 and upregulate LRG1 expression in hippocampal. Furthermore, increased LRG1 expression activates TGF‐β signaling and promotes synaptic and memory deficits (Figure S6). Manipulation of DNMT3a or LRG1 would provide novel insights into the gene regulatory mechanisms that underlie learning and memory decline after surgery and provide therapeutic targets for PND.

DNA methylation alterations are also present in many neurodegenerative diseases (Migliore and Coppedè 2022). DNA methylation modification patterns, such as stress stimuli or drug exposure, are altered in many conditions. This is consistent with our finding in animal experiments showing a significant reduction in DNA methylation levels in the hippocampal of aged mice. This evidence strongly suggests the critical role of DNA methylation alterations in aging‐related degenerative diseases, including PND. Brain DNA methylation homeostasis is carefully regulated by various factors, including methyltransferases and demethyltransferases (Jones 2012). In this study, we found a significant decrease in the level of DNMT3a, which may be responsible for the decrease in DNA methylation levels after surgery. Notably, other methyltransferases, DNMT1 and demethyltransferase TET2, showed insignificant changes. Ample evidence has indicated that expression of DNMT3a is more susceptible to environmental factors, stimuli, and aging (Sanacora, Yan, and Popoli 2022). Repeated exposure to anesthesia drugs could cause a decrease in DNMT3a expression in neurons, which is associated with addiction behavior. Stress stimuli could also decrease DNMT3a content (Warhaftig et al. 2021). Consequently, we speculated that surgical stimuli and anesthetic drug exposure may explain the reduction in DNMT3a levels after surgery.

Studies have shown that DNMT3a is not simply a critical factor for controlling the morphological of newborn neurons, but regulates adult hippocampal neurogenesis (Zocher et al. 2021). DNMT3a deficiency results in reduced frequency and amplitude of miniature excitatory postsynaptic currents (Khazaei et al. 2023). In this study, we found that the loss of DNMT3a expression results in decreased dendritic complexity and spine maturation in aged mice, whereas DNMT3a overexpression in vivo rescues these abnormalities. However, a recent study found elevated DNMT3a levels in rat brains after anesthesia (Fan, Shi, and Zhao 2021). We propose that this discrepancy is due to the age of the animals. The expression of DNMT3a in brain would decrease with aging (Oliveira, Hemstedt, and Bading 2012). More importantly, we found that DNMT3a overexpression in neurons is sufficient to reverse the downregulation of DNA methylation and cognitive dysfunction after surgery in aged mice, providing the important evidence for the role of DNMT3a and DNA methylation in mediating postoperative behavioral phenotypes. Given the important roles of DNMT3a, targeting DNMT3a may be a promising therapeutic approach for PND.

The impact of anesthesia and surgery on DNA methylation patterns, such as the specific genes and pathways, in the aging brain remains elusive. Using RRBS, we found numerous hyper or hypomethylated DMRs across the genome. This is consistent with previous observations that both directions of epigenetic alterations usually occur across the genome when the corresponding catalyzing enzyme is altered (Moyon et al. 2016). When we overlaid the different expression genes and different methylated genes, we found that transcriptional changes are closely linked with alterations in DNA methylation. GO analysis further confirmed the downregulation of synapse organization and upregulation of cell apoptosis. This coincides with the decreased DNMT3a function. By performing a DNMT3a knockout association study (Zocher et al. 2021), we found an association between DNMT3a and LRG1. Decreased DNMT3a may participate in LRG1 upregulation in the hippocampus. Overexpression of DNMT3a restored the LRG1 expression in the hippocampus, whereas mimicking the surgery‐induced decrease in DNMT3a expression increased LRG1 expression. Our in vitro N2a culture studies further demonstrated that DNMT3a directly regulated LRG1 expression. It is well‐known that transcriptional regulation by DNMT3a has pleiotropic effects; however, how DNMT3a specifically targets LRG1 remains unknown. Further study indicated that levels of DNA methylation significantly decreased at chr17:56123031–56123302 in promote of LRG1 after surgery. Chip‐qPCR indicated that these decreases may be related to the reduced binding of DNMT3a to LRG1 promoter region. Overexpression of DNMT3a abolished the decline in LRG1 DNA methylation and decreased LRG1 transcription and protein expression.

The role of LRG1 in promoting angiogenesis was first reported through the activation of the TGF‐β signaling (Wang et al. 2013). An increasing number of publications have reported the involvement of LRG1 in multiple human conditions, including cancer, cardiovascular disease, and neurological disease (Camilli et al. 2022). Stroke or TBI could promote neuronal apoptosis by activating the TGF‐β signaling, and inhibition of LRG1 exerts a neuroprotective effect (Chen et al. 2024; Miao et al. 2022). The latest research suggests that LRG1 is also highly expressed in neurons (Ruan et al. 2023). High levels of LRG1 have been implicated in cognitive dysfunction (Low et al. 2024). fEPSPs were significantly smaller in LRG1‐overexpressing mice, indicating impaired synaptic transmission in LRG1, and these differences became evident with age (Akiba et al. 2017). Our in vivo experiments further confirmed the remedial effects of LRG1 silencing on dendritic abnormalities and synaptic/memory deficits. They reversed memory impairment after surgery, supporting the hypothesis that increased LRG1 firing plays a causal role in PND. We also identified downstream targets of LRG1, TGF‐β signaling. As a pleiotropic cytokine, TGF‐β was generally recognized as an anti‐inflammatory factor. However, numerous studies have shown that TGF‐β significantly increases in injured brains or during LPS‐induced neuroinflammation, leading to hyperactivation of the TGF‐β signaling. Such hyperactivation was associated with neuronal apoptosis and impaired synaptic function (Miao et al. 2022; Patel et al. 2017; Rong et al. 2023). The role of TGF‐β in PND has also been reported. Anesthesia and surgery can activate TGF‐β signaling, exacerbating early cell apoptosis (Shen et al. 2021; Sun et al. 2020). These findings are consistent with our results. In addition, we found that manipulation of DNMT3a or LRG1 can reverse changes in the TGF‐β signaling. These results further verify that DNMT3a and LRG1 may be key factors mediating the development of PND. However, the roles of different downstream molecules in the TGF‐β pathway in PND require further exploration.

This study has some limitations. We speculate that LRG1 is not the sole downstream target of DNMT3a in PND. Some other different methylated genes are also related to the disruption of neuronal morphology remodeling and learning memory. Whether the expression of these genes is dependent on epigenetic modifications after surgery remains elusive.

In conclusion, this study showed that anesthesia/surgery decreases DNMT3a levels and leads to DNA methylation changes and synaptic disorders, at least in part through the epigenetic regulation of LRG1and activates TGF‐β signaling (Figure S6). This may be a key factor mediating the development of postoperative cognitive impairment. These data suggest that DNMT3a represents a new molecular target for treating postoperative cognitive impairment in older patients.

## Author Contributions

Lize Xiong and Peilin Cong designed the experiments. Li Tian and Peilin Cong contributed to writing the manuscript. Xinwei Huang, Qian Zhang, and Mengfan He contributed to performing the experiments. Hanxi Wan and Chun Cheng contributed to the data analysis. Yuxin Zhang, Qianqian Wu, and Huanghui Wu contributed to editing the images. All authors contributed to the article and approved the submitted version.

## Conflicts of Interest

The authors declare no conflicts of interest.

## Supporting information


**Figure S1.** Anesthesia/surgery‐impaired learning and memory in aged mice. (A) Schematic of the experimental paradigm. (B) The average speed, (C) time spent in the central area, and (D) the representative movement tracks in the open‐field tests (*n* = 8). (E) The escape latency over training session. (F) The percentage of time mice spent in the target quadrant and (G) the representative mouse movement tracks (*n* = 8). (H) Percentage of freezing times (*n* = 8). All values are presented as mean ± SEM ( **p < 0.01, and ***p < 0.001, unpaired *t* test for B, C, F, H, and two‐way ANOVA with Bonferroni post hoc test for E) (related to Figure 1).

**Figure S2.** Anesthesia/surgery‐induced differential transcriptional changes on postoperative day 1. (A) Experimental design for long‐term memory on postoperative day 1. (B) The freezing times between surgery group and control group (*n* = 10). (C) Experimental design for short‐term memory on postoperative day 1. (D) The freezing times between surgery group and control group (*n* = 10). (E) PCA plot of gene expression data obtained via RNA‐seq data for three biological replicates corresponding to the samples from the hippocampus in the control and surgery groups. (F) Heatmap summary and hierarchical clustering showed clear differences between samples from the hippocampus in the control and surgery groups.

**Figure S3.** Anesthesia/surgery does not affect 5‐hmC levels in the hippocampus of aged mice. (A) Representative field and quantification of 5‐hmC expression in the hippocampus (*n* = 9). (B) Global DNA methylation level in the hippocampus in the surgery and control groups (*n* = 3). All values are presented as mean ± SEM (unpaired *t* test for A, B).

**Figure S4.** DNMT3a knockdown activates the TGF‐β signaling. (A) Protein blotting bands quantified relative protein expression for TGF‐β1, SMAD2, p‐SMAD2, SMAD3, and p‐SMAD3 in EGFP‐ and shDNMT3a AAV‐injected mice (*n* = 4). (B) Protein blotting bands quantify relative protein expression for TGF‐β, SMAD2, p‐SMAD2, SMAD3, and p‐SMAD3 between the surgery and control groups (*n* = 4). All values are presented as mean ± SEM (*p < 0.05, **p < 0.01, and ***p < 0.001, unpaired *t* test for B, D).

**Figure S5.** Blocking hippocampus LRG1 rescues anesthesia/surgery‐induced memory impairment and synaptic disorder in aged mice. (A) Schematic of the experimental paradigm and diagram of the AAV. (B) Representative fluorescence image of the virus‐infected slice. (C) Protein blotting bands for LRG1. (D) Quantification of relative protein expression. (E) Relative gene expression of LRG1 in hippocampus extracts. (F) Protein blotting band quantification of relative protein expression for TGF‐β1, SMAD2, p‐SMAD2, SMAD3, and p‐SMAD3 (*n* = 4). (G) The normalized fEPSP slope and amplitude at hippocampal. Quantitative analysis of fEPSP slope and amplitude in the last 20 min (*n* = 5). (H) The average speed, (I) time spent in the central area, and (J) representative movement tracks in the open‐field tests (*n* = 8). (K) The escape latency over the training session. (L) The percentage of times mice spent in the target quadrant and (M) representative movement tracks (*n* = 8). (N) The percentage of freezing times (*n* = 8). All values are presented as mean ± SEM (*p < 0.05, **p < 0.01, unpaired *t* test for D, E, F, G, H, I, L, N and two‐way ANOVA with Bonferroni post hoc test for K).

**Figure S6.** Anesthesia/surgery decrease DNMT3a levels and leads to DNA methylation changes and synaptic disorders associated with PND. DNMT3a downregulation appears to causally contribute to surgery‐induced cognitive impairment, whereas its overexpression effectively alleviated cognitive impairment behaviors. Mechanistically, DNMT3a downregulation induced by anesthesia/surgery could disrupt DNA methylation stability in the hippocampus of aged mice. This decreased the binding of DNMT3a to the Lrg1 promoter and upregulated Lrg1 expression in hippocampal neurons. Furthermore, increased Lrg1 expression activates TGF‐β signaling and promotes synaptic and memory deficits.

**Table S1.** List of primers in the article.

**Table S2.** DEGs between surgery and control groups.

**Table S3.** DMRs between surgery and control groups.

**Table S4.** Hypomethylated and upregulated genes.

**Table S5.** Hypermethylated and downregulated genes.

## Data Availability

The data that supports the findings of this study are available in the supplementary material of this article. A portion of the RNA‐seq data relevant to this study was obtained from publicly available sources in the GEO database (https://www.ncbi.nlm.nih.gov/geo/; GSE167953).
