# Correction to “DNMT3a Deficiency Contributes to Anesthesia/Surgery‐Induced Synaptic Dysfunction and Cognitive Impairment in Aged Mice”

**DOI:** 10.1111/acel.70611

**Published:** 2026-06-25

Cong, P. , X. Huang , Q. Zhang , et al. 2025. “DNMT3a Deficiency Contributes to Anesthesia/Surgery‐Induced Synaptic Dysfunction and Cognitive Impairment in Aged Mice.” Aging Cell 24: e14458. 10.1111/acel.14458.39722450 PMC11984699

In Figure 3I, the *x*‐axis group labels were missing below the rightmost fEPSP amplitude quantification bar graph, and the EGFP and shDnmt3a groups in this bar graph were mistakenly labeled with incorrect colors during figure assembly. The corrected image is provided here. This correction does not affect the underlying data, statistical analysis, interpretation of the results, or conclusions of the study.

**FIGURE 3:**
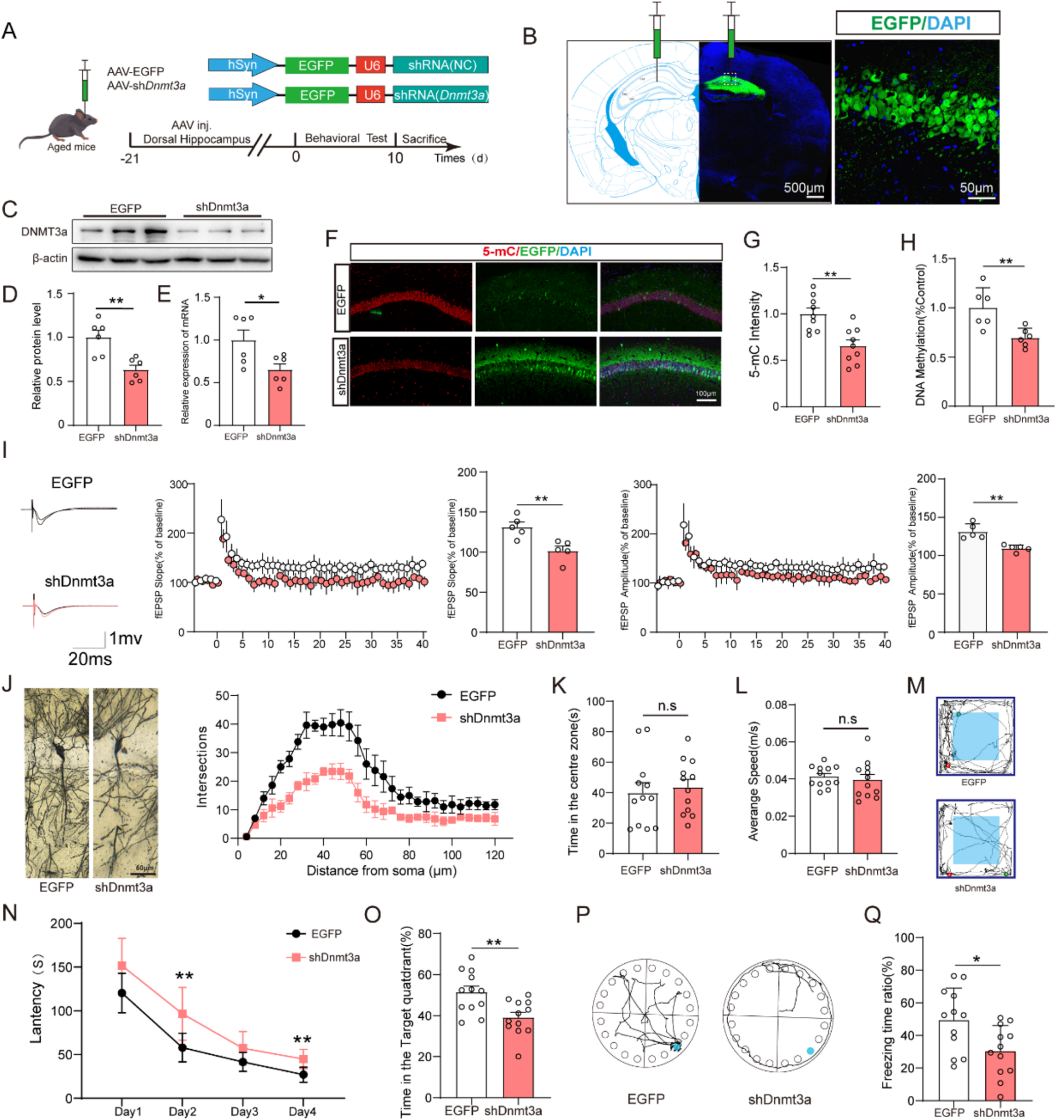
DNMT3a knockdown in the hippocampus leads to decreased DNA methylation levels, synaptic disorder, and memory deficiency. (A) Schematic of the experimental paradigm. (B) Representative fluorescence image of the AAV‐infected slice. (C) Protein blotting bands for DNMT3a. (D) Quantification of relative protein expression. (E) Relative gene expression in hippocampus (*n* = 6). (F, G) Representative field and quantification of 5‐mC expression in the CA1 (*n* = 9). (H) Global DNA methylation level in the hippocampus (*n* = 6). (I) Normalized the fEPSP slope and amplitude at hippocampal. Quantitative analysis of fEPSP slope and amplitude at last 20 min (*n* = 5). (J) Golgi staining images showing the dendritic trees in hippocampus. The Sholl analysis was performed to evaluate the dendritic complexity (*n* = 5). The average speed (K), time spent in the central area (L), and representative movement track (M) in OFT (*n* = 12). The escape latency over the training session (N). The percentage of times spent in the target quadrant (O) and representative movement tracks (P) (*n* = 12). (Q) The percentage of freezing times (*n* = 12). All values are presented as mean ± SEM (*p < 0.05, **p < 0.01, unpaired *t*‐test for D, E, G, H, I, K, L, O, Q and two‐way ANOVA with Bonferroni post hoc test for J).

We apologize for this error.

