# Supplementary figures and images for: DNMT3a Deficiency Contributes to Anesthesia/Surgery‐Induced Synaptic Dysfunction and Cognitive Impairment in Aged Mice
Source: Aging Cell. 2024 Dec 25;24(4):e14458. doi: 10.1111/acel.14458 (PMC11984699; doi:10.1111/acel.14458)

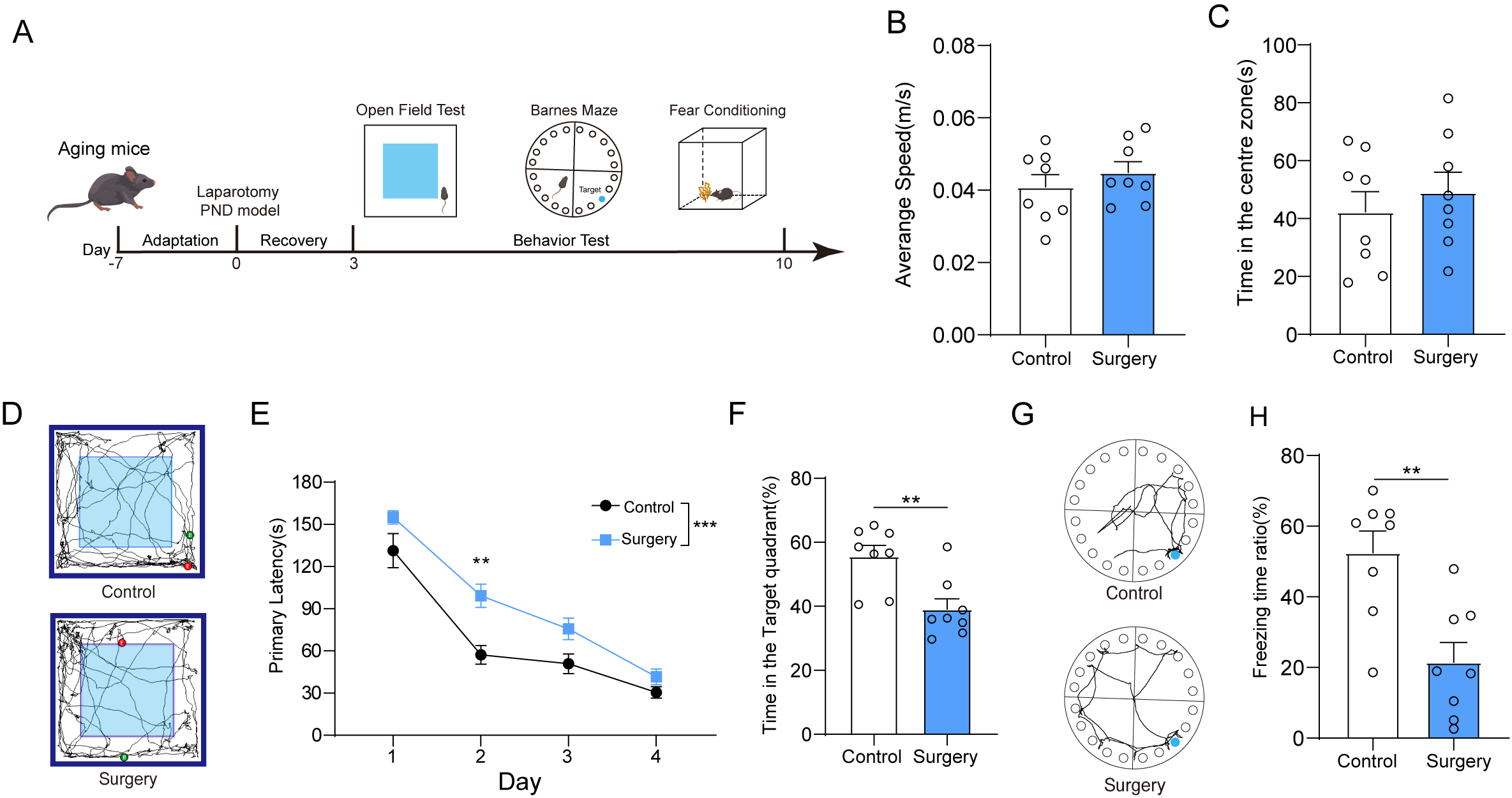

Supplement: Supplementary file 1 — Figure S1. Anesthesia/surgery‐impaired learning and memory in aged mice. (A) Schematic of the experimental paradigm. (B) The average speed, (C) time spent in the central area, and (D) the representative movement tracks in the open‐field tests (n = 8). (E) The escape latency over training session. (F) The percentage of time mice spent in the target quadrant and (G) the representative mouse movement tracks (n = 8). (H) Percentage of freezing times (n = 8). All values are presented as mean ± SEM ( **p < 0.01, and ***p < 0.001, unpaired t test for B, C, F, H, and two‐way ANOVA with Bonferroni post hoc test for E) (related to Figure 1). [file ACEL-24-e14458-s009.tif]

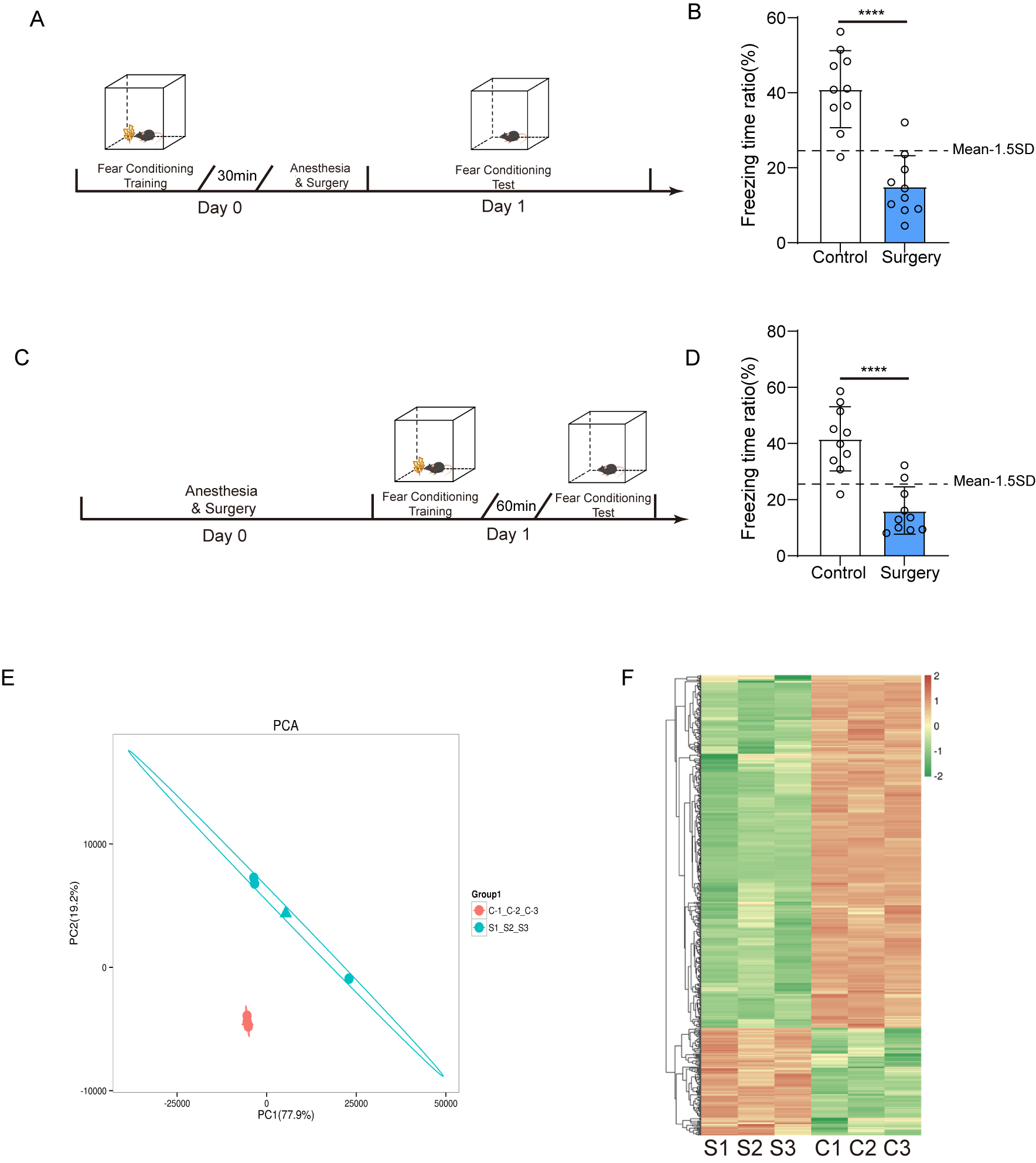

Supplement: Supplementary file 2 — Figure S2. Anesthesia/surgery‐induced differential transcriptional changes on postoperative day 1. (A) Experimental design for long‐term memory on postoperative day 1. (B) The freezing times between surgery group and control group (n = 10). (C) Experimental design for short‐term memory on postoperative day 1. (D) The freezing times between surgery group and control group (n = 10). (E) PCA plot of gene expression data obtained via RNA‐seq data for three biological replicates corresponding to the samples from the hippocampus in the control and surgery groups. (F) Heatmap summary and hierarchical clustering showed clear differences between samples from the hippocampus in the control and surgery groups. [file ACEL-24-e14458-s007.tif]

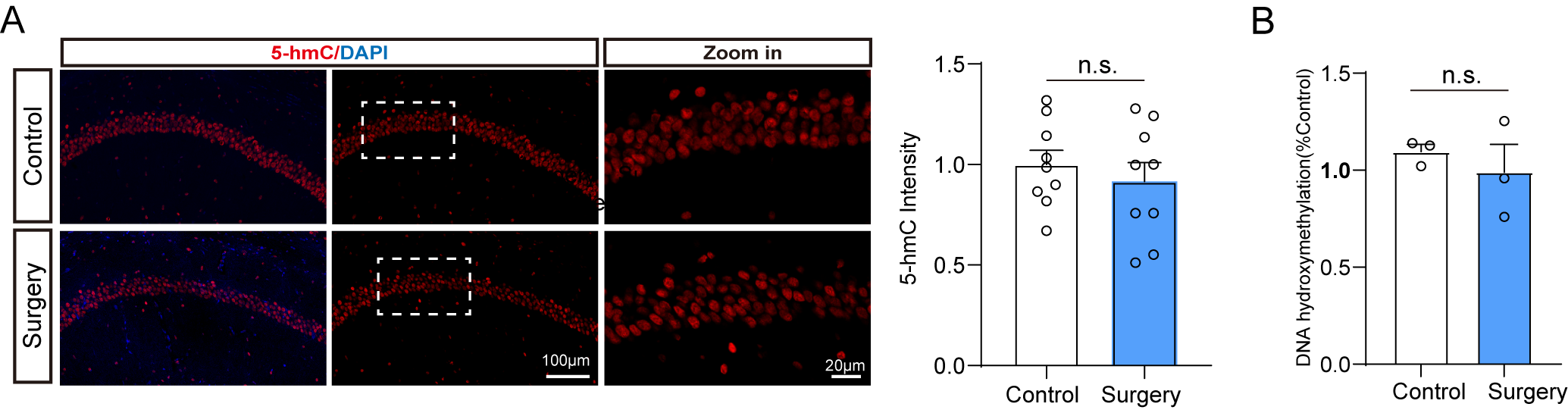

Supplement: Supplementary file 3 — Figure S3. Anesthesia/surgery does not affect 5‐hmC levels in the hippocampus of aged mice. (A) Representative field and quantification of 5‐hmC expression in the hippocampus (n = 9). (B) Global DNA methylation level in the hippocampus in the surgery and control groups (n = 3). All values are presented as mean ± SEM (unpaired t test for A, B). [file ACEL-24-e14458-s003.tif]

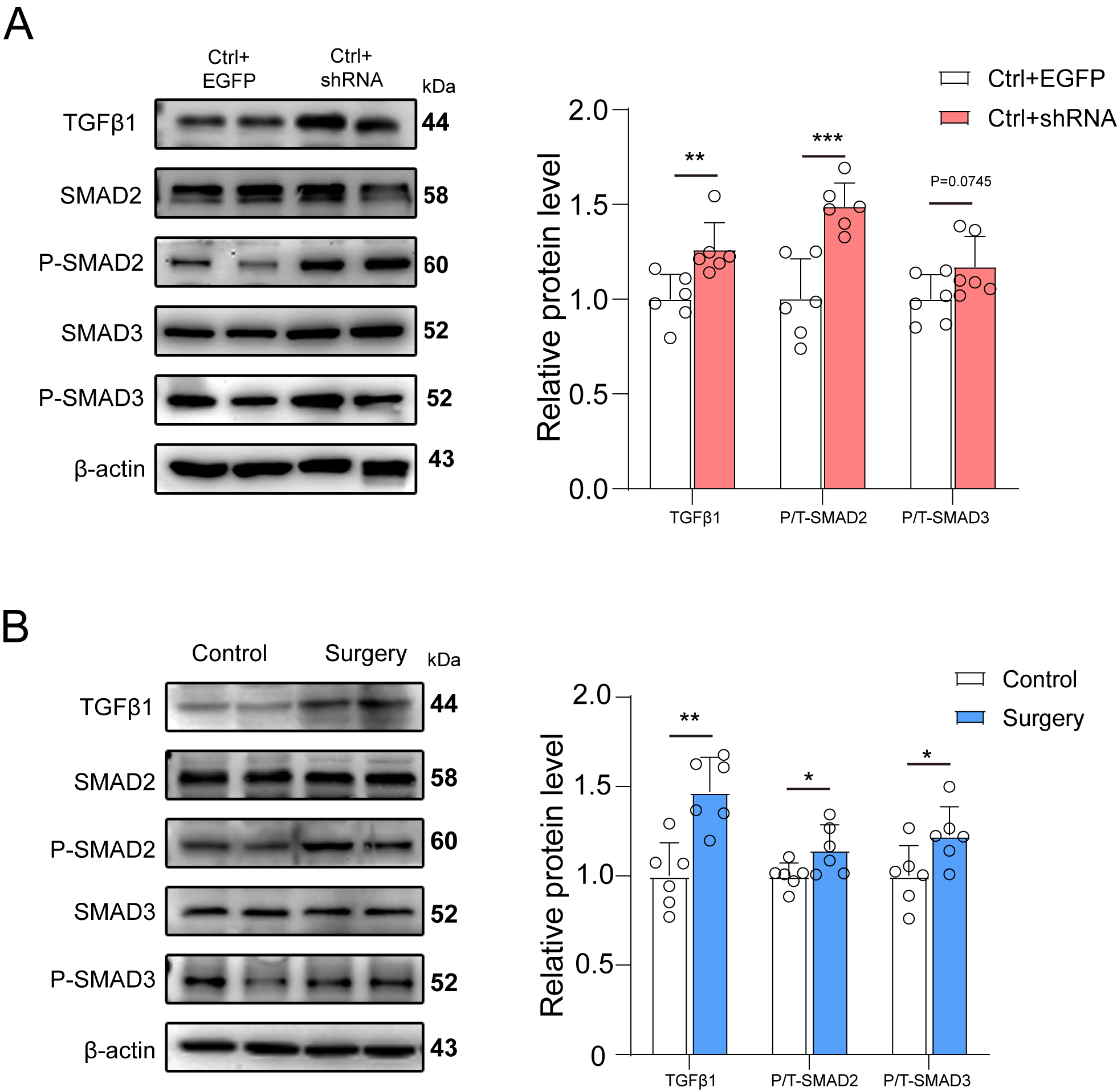

Supplement: Supplementary file 4 — Figure S4. DNMT3a knockdown activates the TGF‐β signaling. (A) Protein blotting bands quantified relative protein expression for TGF‐β1, SMAD2, p‐SMAD2, SMAD3, and p‐SMAD3 in EGFP‐ and shDNMT3a AAV‐injected mice (n = 4). (B) Protein blotting bands quantify relative protein expression for TGF‐β, SMAD2, p‐SMAD2, SMAD3, and p‐SMAD3 between the surgery and control groups (n = 4). All values are presented as mean ± SEM (*p < 0.05, **p < 0.01, and ***p < 0.001, unpaired t test for B, D). [file ACEL-24-e14458-s004.tif]

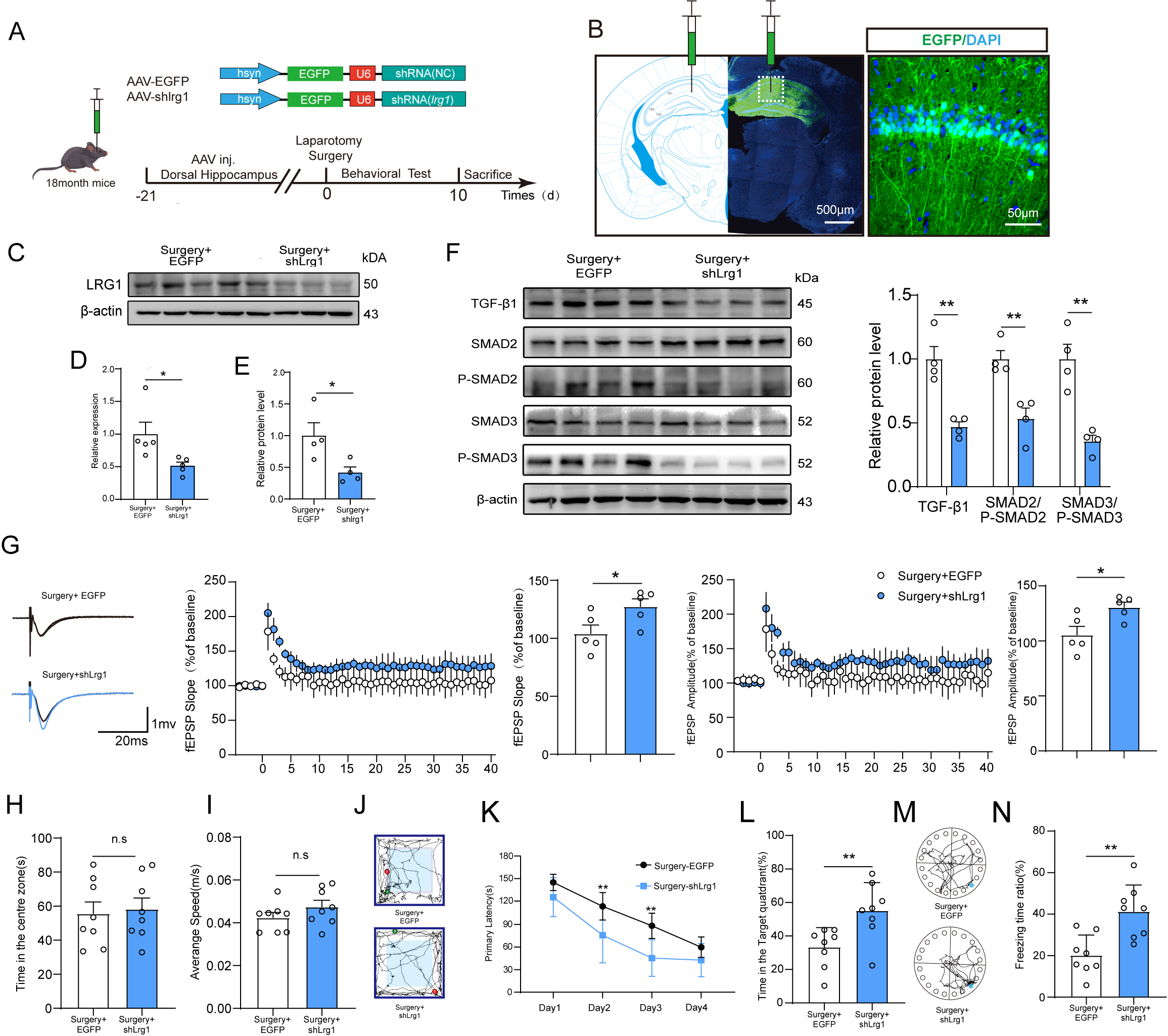

Supplement: Supplementary file 5 — Figure S5. Blocking hippocampus LRG1 rescues anesthesia/surgery‐induced memory impairment and synaptic disorder in aged mice. (A) Schematic of the experimental paradigm and diagram of the AAV. (B) Representative fluorescence image of the virus‐infected slice. (C) Protein blotting bands for LRG1. (D) Quantification of relative protein expression. (E) Relative gene expression of LRG1 in hippocampus extracts. (F) Protein blotting band quantification of relative protein expression for TGF‐β1, SMAD2, p‐SMAD2, SMAD3, and p‐SMAD3 (n = 4). (G) The normalized fEPSP slope and amplitude at hippocampal. Quantitative analysis of fEPSP slope and amplitude in the last 20 min (n = 5). (H) The average speed, (I) time spent in the central area, and (J) representative movement tracks in the open‐field tests (n = 8). (K) The escape latency over the training session. (L) The percentage of times mice spent in the target quadrant and (M) representative movement tracks (n = 8). (N) The percentage of freezing times (n = 8). All values are presented as mean ± SEM (*p < 0.05, **p < 0.01, unpaired t test for D, E, F, G, H, I, L, N and two‐way ANOVA with Bonferroni post hoc test for K). [file ACEL-24-e14458-s005.tif]

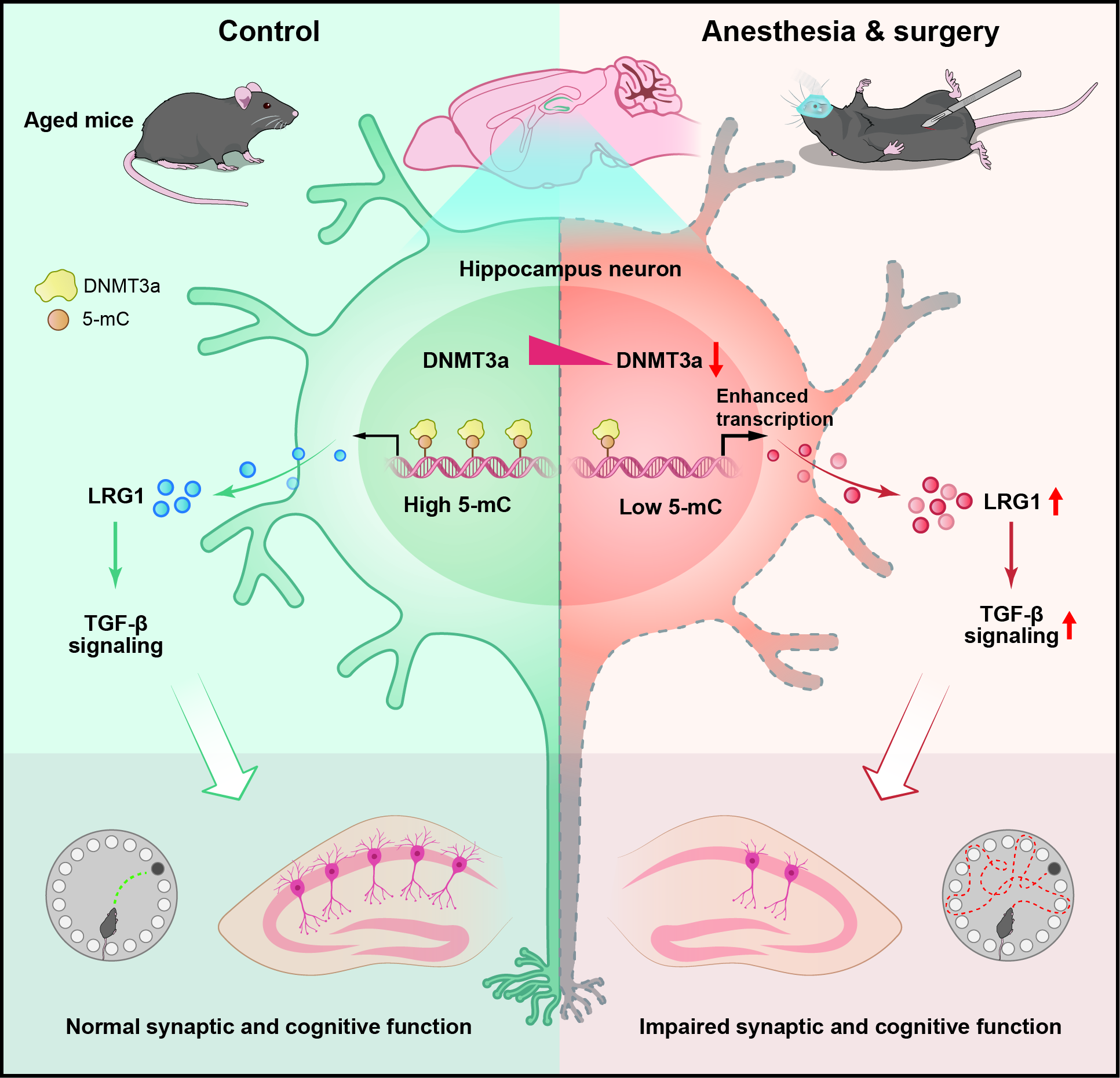

Supplement: Supplementary file 6 — Figure S6. Anesthesia/surgery decrease DNMT3a levels and leads to DNA methylation changes and synaptic disorders associated with PND. DNMT3a downregulation appears to causally contribute to surgery‐induced cognitive impairment, whereas its overexpression effectively alleviated cognitive impairment behaviors. Mechanistically, DNMT3a downregulation induced by anesthesia/surgery could disrupt DNA methylation stability in the hippocampus of aged mice. This decreased the binding of DNMT3a to the Lrg1 promoter and upregulated Lrg1 expression in hippocampal neurons. Furthermore, increased Lrg1 expression activates TGF‐β signaling and promotes synaptic and memory deficits. [file ACEL-24-e14458-s001.tif]
